# Identification of endothelial-related molecular subtypes for bladder cancer patients

**DOI:** 10.3389/fonc.2023.1101055

**Published:** 2023-03-21

**Authors:** Deng-xiong Li, De-chao Feng, Xu Shi, Rui-cheng Wu, Kai Chen, Ping Han

**Affiliations:** Department of Urology, Institute of Urology, West China Hospital, Sichuan University, Sichuan, Chengdu, China

**Keywords:** bladder cancer, endothelial cell, tumor immune environment, single-cell RNA-sequencing, immunotherapy

## Abstract

**Background:**

Bladder cancer (BC) is a disease with significant heterogeneity and poor prognosis. The prognosis and therapeutic response of BC patients are significantly influenced by endothelial cells in the tumor microenvironment. In order to understand BC from the perspective of endothelial cells, we orchestrated molecular subtypes and identified key genes.

**Methods:**

Single-cell and bulk RNA sequencing data were extracted from online databases. R and its relative packages were used to analyze these data. Cluster analysis, prognostic value analysis, function analysis, immune checkpoints, tumor immune environment and immune prediction were conducted.

**Results:**

Five endothelial-related genes (CYTL1, FAM43A, HSPG2, RBP7, and TCF4) divided BC patients in the TCGA, GSE13507, and GSE32894 datasets into two clusters, respectively. In prognostic value analysis, patients in the cluster 2 were substantially associated with worse overall survival than those in the cluster 1 according to the results of TCGA, GSE13507 and GSE32894 datasets. In the results of functional analysis, the endothelial-related clusters was enriched in immune-related, endothelial-related and metabolism-related pathways. Samples in the cluster 1 had a statistically significant increase in CD4+ T cells and NK-cell infiltration. Cluster 1 was positively correlated with the cancer stem score and tumor mutational burden score. The results of immune prediction analysis indicated that 50.6% (119/235) of patients in the cluster 1 responded to immunotherapy, while the response rate in the cluster 2 decreased to 16.7% (26/155).

**Conclusion:**

In this study, we categorized and discovered distinctive prognosis-related molecular subtypes and key genes from the perspective of endothelial cells at the genetic level by integrating single-cell and bulk RNA sequencing data, primarily to provide a roadmap for precision medicine.

## Introduction

Bladder cancer (BC) ranks as the 6th most common cancer and the 9th leading cause of cancer death in men (1). The mortality rate of BC patients remains high in developing countries, although the prognosis for BC patients in developed counties has significantly improved through various treatment options (including surgery, chemotherapy, and immunotherapy) (1). While suffering a great physical, mental, and financial load, many patients do not have an improved prognosis and only a few patients can benefit from these therapies (2). For instance, radical cystectomy will cause serious harm and drastically lower the quality of daily life. Unfortunately, postoperative survival outcomes are dismal. Therefore, these improvements are far from satisfactory. To provide a solution to this conundrum, researchers are striving to understand the mechanism of BC to prevent the occurrence of BC. We also hope to identify key genes that could pave the way for new, efficient medications (such as anti-PD1/-L1). In addition to exploring new mechanisms and targets, how to apply current treatments is also a field of great concern. To solve this problem, physicians search for highly efficient markers, such as the neutrophil-to-lymphocyte ratio (3) and histological features (4), to choose the optimal therapy for each patient. Researchers are also selecting genes with prognostic value to aid doctors in clinical decisions (5).

An organized microvasculature consists of an endothelial cell layer supported by a basement membrane and surrounded by perivascular cells (pericytes) that provides nourishment for the urothelium as well as its underlying lamina propria and muscularis propria (6, 7). In contrast, in tumor tissue, endothelial cells are surrounded by fewer pericytes, which leads to leaky inter-endothelial cell junctions (8). Hypoxia and nutrient deprivation brought on by leaky tumor vasculature enhance the invasive properties of BC cells (9). Due to the different degrees of leaky tumor vasculature, BC patients have considerably variable survival outcomes (10). Furthermore, tumor cells farther from the vasculature are exposed to lower drug concentrations than those tumor cells closer to it, resulting in differences in treatment outcomes (11). Meanwhile, an insufficient nutrient supply and hypoxia induced by endothelial cells can also promote the enrichment of cancer stem cells, which is similarly related to treatment outcomes (12). Endothelial cell can cause treatment resistance and therefore significantly affect the prognosis and of BC. Recently, single-cell RNA-sequencing (scRNA-seq) technologies have attracted wide attention and allowed us to sequence and analyze thousands of cells per tumor. This scale allows it to discriminate different cell subsets, enabling researchers to explore the function of specific cell infiltration. For instance, immunotherapy relies heavily on the tumor environment. Immunotherapy may be more effective for patients with high CD8 T-cell infiltration than for those with low CD8 T-cell infiltration (13). Although endothelial cells are crucial for BC, there are no endothelial-molecular subtypes which exhibits prognostic value in BC.

Thus, in this study, we collected and analyzed BC data from online databases, integrating single-cell and bulk RNA sequencing data, in order to orchestrate molecular subtypes and identify key genes for BC from the perspective of endothelial cells, paving the way for the development of precision medicine. Then, various methods were employed to validate the cluster and key genes.

## Materials and methods

### Data acquisition

Through analysis of the single-cell data shared in publications, the Tumor Immunotherapy Gene Expression Resource (http://tiger.canceromics.org/#/home, TIGER) provides cell type markers for free. In this study, we downloaded the markers of endothelial cells with |log2FoldChange| >0.3 based on a prior work (14). BC and normal tissue data, including expression data and clinical data, were extracted from the Cancer Genome Atlas (www.gdc.cancer.Gov, TCGA ). The ‘limma’ package was used to identify the differentially expressed genes between 19 normal samples and 414 BC samples. The differentially expressed genes should have a P value <0.05 and |log2FoldChange| >1. Furthermore, in terms of clinical analysis, patients were disqualified if they had any of the following features: postoperative survival time less than 30 days, not a pure transitional cell cancer, or no survival outcome. The endothelial cell content of each included BC sample in the TCGA dataset was determined with the EPIC algorithm in the ‘immuneeconv’ package (https://github.com/immunomind/immunarch). Then, the correlation between the endothelial cell content and genes from the TCGA dataset was assessed by Pearson correlation analysis. The endothelial-related genes should have |coefficients| > 0.3 and P value < 0.05. To improve the credibility of the results, we validated the results in two external datasets GSE13507 and GSE32894 which were retrieved from the Gene Expression Omnibus (https://www.ncbi.nlm.nih.gov, GEO) database.

### Molecular subtypes

After acquiring the aforementioned data, co-expressed genes were screened by the ‘VennDiagram’ package. Then, all co-expressed genes were incorporated into the lasso regression model, which could apply penalties to these genes. The penalty parameter (λ) for the model was determined by 10-fold cross-validation following the minimum criteria. A total of seven genes were selected by lasso regression analysis. The results of the univariable Cox regression model indicated that only five of these genes had significant prognostic value. Then, the patients in the TCGA, GSE13507, and GSE32894 datasets were subtyped by the R packages “ConsensusClusterPlus” and “limma” using the five genes (CYTL1, FAM43A, HSPG2, RBP7, and TCF4). The consensus matrix k value denoted the number of endothelial-related clusters (ERC).

### Evaluation of the cluster and function analysis

At first, we assessed the prognostic value of endothelial cell based on the basis of the outcomes of EPIC algorithm and TCGA dataset. To validate the prognostic value of endothelial cell, we also employed MCPCOUNTER algorithm (https://github.com/ebecht/MCPcounter) to calculate the endothelial cell content of each included patients in TCGA dataset. Then, the prognostic value of endothelial cell was identified by Kaplan−Meier curves. After establishing the cluster, Kaplan−Meier curves were utilized to estimate the prognostic value of ERC. First, Kaplan−Meier curves were employed to analyze whether the ERC could predict the overall survival (OS) of patients in the TCGA, GSE13507 and GSE32894 datasets. Meanwhile, the prognostic value of ERC was also validated in various clinical subgroups. Then, according to the results of univariable Cox regression model, covariates with a P value <0.1 were included in the multivariable Cox regression analysis to estimate the independent prognostic value of ERC.

Based on the TCGA dataset and ERC, we performed Gene Ontology (GO) enrichment analysis, which consisted of molecular function (MF), biological process (BP) and cellular component (CC). The following criteria were used to choose the GO terms in light of the GO results: P value < 0.05 and Q < 0.05. Similarly, the enriched Kyoto Encyclopedia of Genes and Genomes (KEGG) pathways were screened out with a P value < 0.05 and Q <0.05. For further evaluation of the potential pathways, REACTOME pathways were carefully selected by Gene Set Enrichment Analysis (GSEA) with a P value < 0.05 and FDR<25%. Furthermore, the interacting proteins of CYTL1, FAM43A, HSPG2, RBP7, and TCF4 were screened by GeneMANIA (www.genemania.org) (15). Furthermore, Tumor Immune Single-cell Hub 2 (http://tisch.comp-genomics.org, TISH2) (16) collects tumor-related scRNA-seq from human studies, providing the differentially expressed genes between endothelial cell and other infiltrating cells based on the scRNA-seq. The function of endothelial cells was next investigated using GO and KEGG analyses with selective criteria P value < 0.05 and Q < 0.05. As is common knowledge, transcription factors are deemed as promising targets for innovative new drugs. Thus, TISH2 also utilized to identify the interacting transcription factors of endothelial cell based on scRNA-seq.

### Immune-related analysis

Immune-related analysis was adopted because the functional results indicated that the ERC is mainly engaged in immune-related activities. To identify the correlation between the ERC and immune checkpoints, we compared the expression of 32 checkpoints between the clusters and plotted them as bar plots. Then, we compared the immune cells that had penetrated the clusters in EPIC.

Following our investigation of tumor microenvironment, we obtained tumor mutational burden (TMB) data from the TCGA dataset. Then, the overall TMB was calculated by the ‘maftools’ package and the TMB scores of the clusters were compared. Moreover a mRNA expression‐based stemness index (mRNAsi) was established by a one-class logistic regression machine learning algorithm (17). The mRNAsi can reflect the expression of the tumor immune microenvironment and predict the immunotherapy response. Therefore, we also calculated and compared the mRNAsi score between the clusters. To further verify the above results, based on the TCGA-BC cohort, the Tumor Immune Dysfunction and Exclusion (TIDE) (18) algorithm was used to predict and compare the immunotherapy response between the cluster 1 and cluster 2.

### Statistical analysis

According to the normality and quality of variances of the data, one-way ANOVA or the Mann−Whitney U test was used to perform statistical analysis of three or more continuous variables. Quantitative data in two groups were compared using Student’s t test. All analyzed data are displayed as the standard deviation (SD). A P<0.05 was considered significant for all analyses, which were performed using R version 3.6.3 and relative packages. ns, P≥0.05; *, P< 0.05; **, P<0.01; ***, P<0.001.

## Results

### Construction of the cluster and basic data

**Figure 1:**
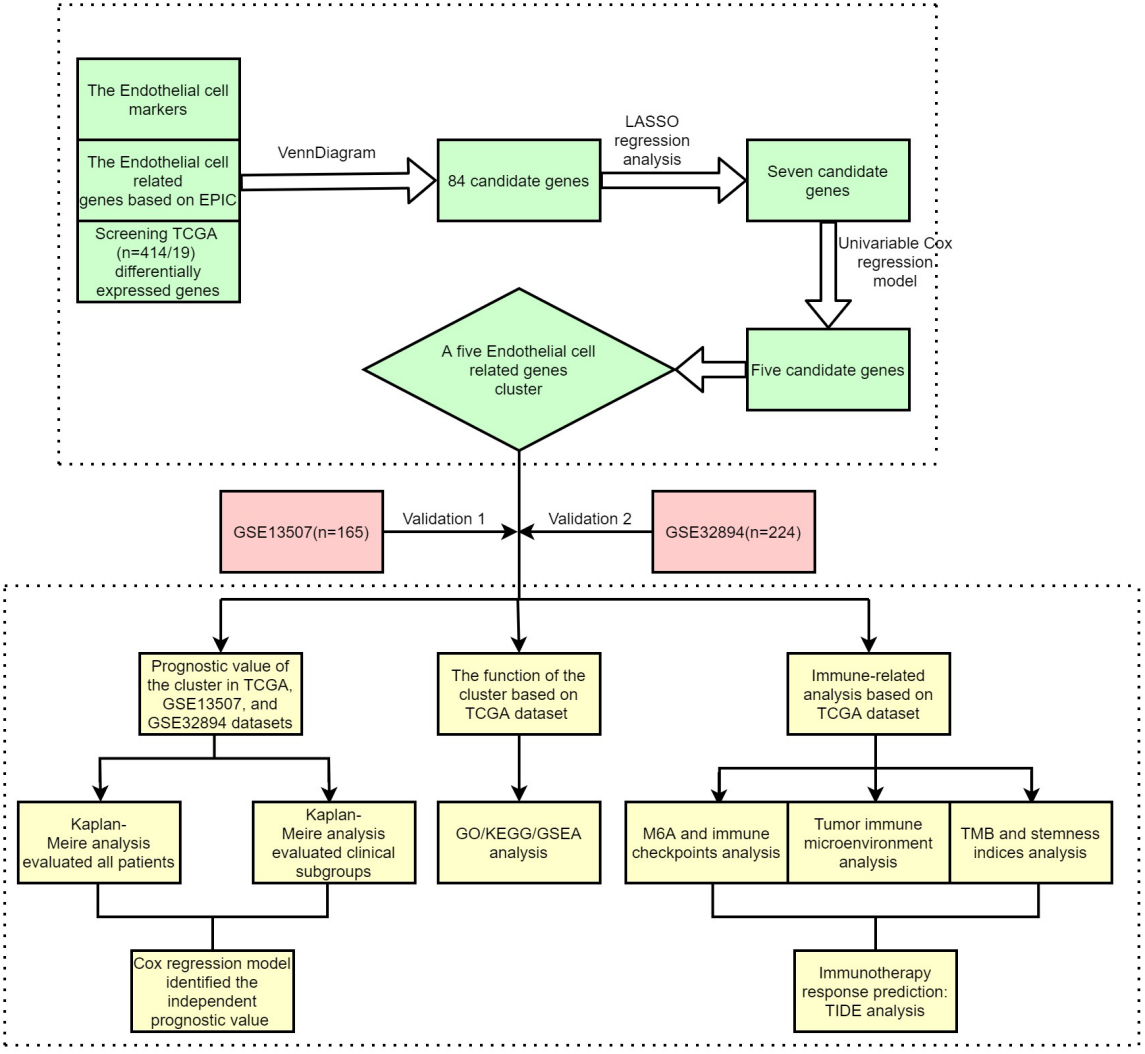
The workflow of this study.

**Figure 2:**
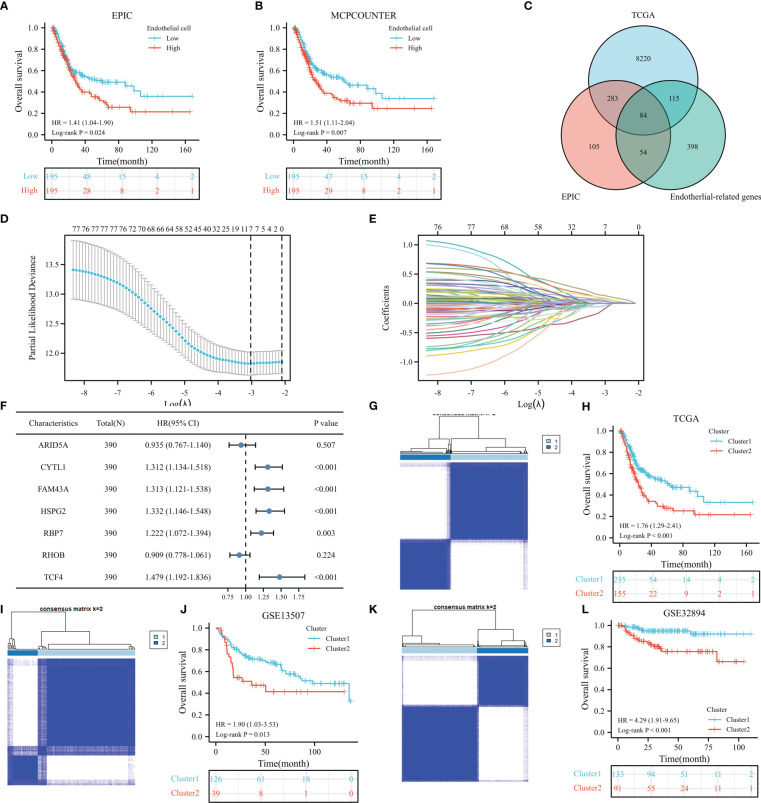
The prognostic value of endothelial cells based on the results of EPIC **(A)** and MCPCOUNTER algorithms **(B)**, the co-expressed genes **(C)**, the cross-validation to determine the optimal penalty parameter lambda **(D)**, Lasso regression of the seven endothelial cell-related genes **(E)**, the prognostic value of these seven genes in overall survival (OS) according to the results of univariable Cox regression analysis in TCGA dataset **(F)**, cluster plot showing two distinct groups in the TCGA database **(G)**, the Kaplan−Meier analysis results of OS in TCGA dataset **(H)**, cluster plot showing two distinct groups in the GSE13507 database **(I)**, the Kaplan−Meier analysis results of OS in GSE13507 dataset **(J)**, cluster plot showing two distinct groups in the GSE32894 database **(K)**, the Kaplan−Meier analysis results of OS in GSE32894 dataset **(L)**.

**Figure 1** shows the workflow of our study. **Supplementary Table 1** lists the endothelial-related genes based on the EPIC. Kaplan−Meier curves revealed that patients with high endothelial cell infiltration were significantly associated with worse OS than those with low endothelial cell infiltration based on the results of EPIC (**Figure 2A**, P=0.024) and MCPCOUNTER algorithms (**Figure 2B**, P=0.007). Final selection comprised 84 significantly co-expressed endothelial-related genes (**Figure 2C**). Then, as shown in **Figures 2D, E**, seven genes were screened by lasso regression analysis. Five genes (CYTL1, FAM43A, HSPG2, RBP7, and TCF4) exhibited significant prognostic value in BC patients according to the results of the univariable Cox regression model (**Figure 2F**). Finally, these five endothelial-related genes divided BC patients in the TCGA dataset into two clusters (**Figure 2G**, consensus matrix k = 2). Patients in the cluster 2 were considerably correlated with poorer OS than patients in the cluster 1 (**Figure 2H**, P<0.001). Similarly, these five endothelial-related genes also classified patients in GSE13507 (**Figure 2I**, consensus matrix k = 2) and GSE32894 (**Figure 2K**, consensus matrix k = 2) datasets into two clusters. There were fewer patients in each of the three clusters 2 than in cluster 1. The OS of cluster 2 was significantly worse than that of cluster 1 in GSE13507 (**Figure 2J**, P=0.013) and GSE32894 (**Figure 2L**, P<0.001) datasets.

390 BC patients were included in the TCGA dataset following the exclusion procedure. **Table 1** shows that patients in the cluster 2 had a larger proportion of WHO high grade, American Joint Committee on Cancer (AJCC) stage III-IV, T3_4 stage, distant metastasis and death. Detailed information on GSE13507 and GSE32894 datasets is shown in **Supplementary Table 2** and **Supplementary Table 3**.

**Table 1:** The clinicopathological characteristics of the TCGA included patients.

Characteristic	Cluster1	Cluster2	p
n	235	155	
Age, mean ± SD	67.1 ± 10.99	69.1 ± 9.76	0.066
BMI, mean ± SD	26.46 ± 5.49	27.6 ± 5.63	0.067
Sex, n (%)			0.351
Female	57 (14.6%)	45 (11.5%)	
Male	178 (45.6%)	110 (28.2%)	
Smoking history, n (%)			0.381
No	68 (18%)	37 (9.8%)	
Yes	161 (42.7%)	111 (29.4%)	
WHO grade, n (%)			0.005
High grade	216 (55.8%)	153 (39.5%)	
Low grade	17 (4.4%)	1 (0.3%)	
AJCC stage, n (%)			< 0.001
AJCC stage I-II	94 (24.2%)	29 (7.5%)	
AJCC stage III-IV	139 (35.8%)	126 (32.5%)	
T stage, n (%)			< 0.001
T3_4	123 (34.3%)	122 (34%)	
Ta_2	87 (24.2%)	27 (7.5%)	
Lymph node metastasis, n (%)			0.050
N+	64 (18.2%)	60 (17.1%)	
N0	143 (40.7%)	84 (23.9%)	
Distant metastasis, n (%)			0.029
M0	137 (70.3%)	48 (24.6%)	
M1	4 (2.1%)	6 (3.1%)	
Overall survival, n (%)			0.001
Alive	148 (37.9%)	71 (18.2%)	
Dead	87 (22.3%)	84 (21.5%)	

AJCC, American Joint Committee on cancer; BMI, body mass index; SD, Standard deviation; WHO, World Health Organization; n, Number.

### ERC had prognostic value

**Figure 3:**
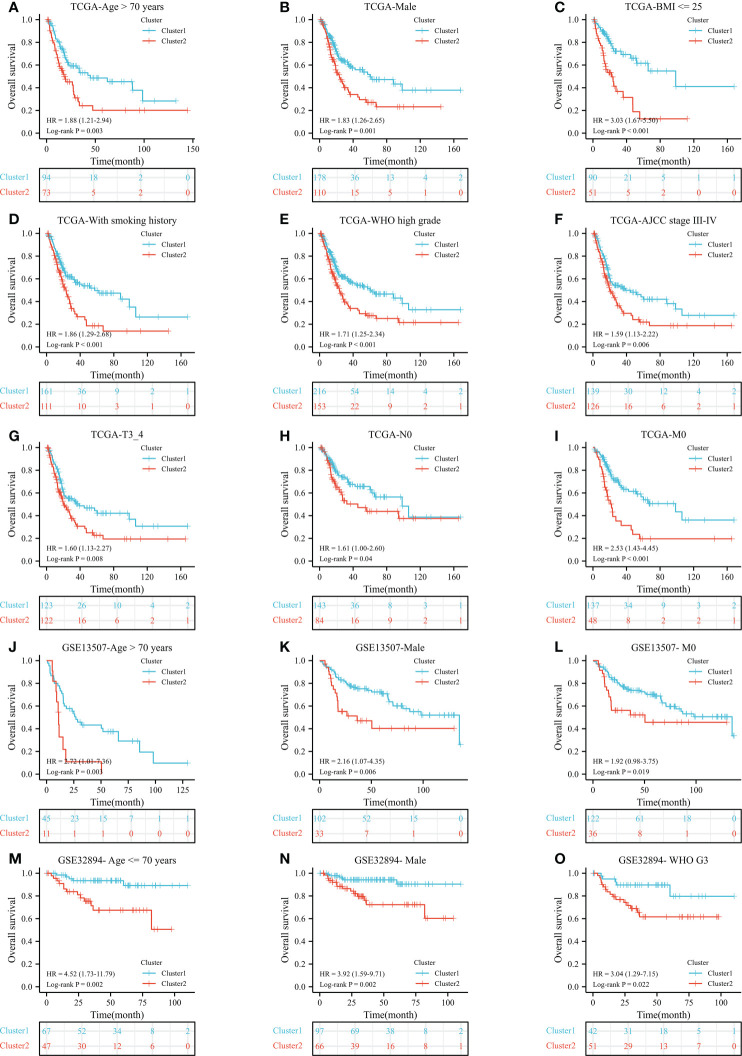
The prognostic ability of the cluster in clinical subgroups: Kaplan−Meier analysis results of subgroups in TCGA dataset: age >70 years **(A)**, male **(B)**, BMI <= 25 **(C)**, with smoking history **(D)**, WHO high grade **(E)**, AJCC stage III-IV **(F)**, T3_4 stage **(G)**, no lymph node metastasis **(H)**, and no distant metastasis **(I)** subgroups; In GSE13507 dataset: age > 70 years **(J)**, male **(K)**, and no distant metastasis **(L)** subgroups; In GSE32894 dataset: age <= 70 years **(M)**, male **(N)**, and WHO G3 **(O)** subgroups. N, lymph node metastasis; M, distant metastasis; WHO, World Health Organization;.

After a preliminary assessment of the prognostic value of ERC, we further verified the prognostic value of the cluster in clinical subgroups. In the TCGA dataset, cluster 2 was significantly associated with worse OS than cluster 1 in many subgroups, such as age >70 years (**Figure 3A**, P=0.003), male sex (**Figure 3B**, P=0.001), body mass index <=25 (**Figure 3C**, P<0.001), smoking history (**Figure 3D**, P<0.001), World Health Organization (WHO) high grade (**Figure 3E**, P<0.001), AJCC stage III-IV (**Figure 3F**, P=0.006), T3_4 stage (**Figure 3G**, P=0.008), no lymph node metastasis (**Figure 3H**, P=0.04) and no distant metastasis (**Figure 3I**, P<0.001) subgroups. Similarly, in the GSE13507 dataset, cluster 2 was statistically correlated with poorer OS than cluster 1 in the age >70 years (**Figure 3J**, P=0.003), male (**Figure 3K**, P=0.006) and no distant metastasis (**Figure 3L**, P=0.019) subgroups. In the GSE32894 dataset, cluster 2 significantly predicted poor OS in the age <=70 years (**Figure 3M**, P=0.002), male (**Figure 3N**, P=0.002) and WHO G3 (**Figure 3O**, P=0.022) subgroups.

**Figure 4:**
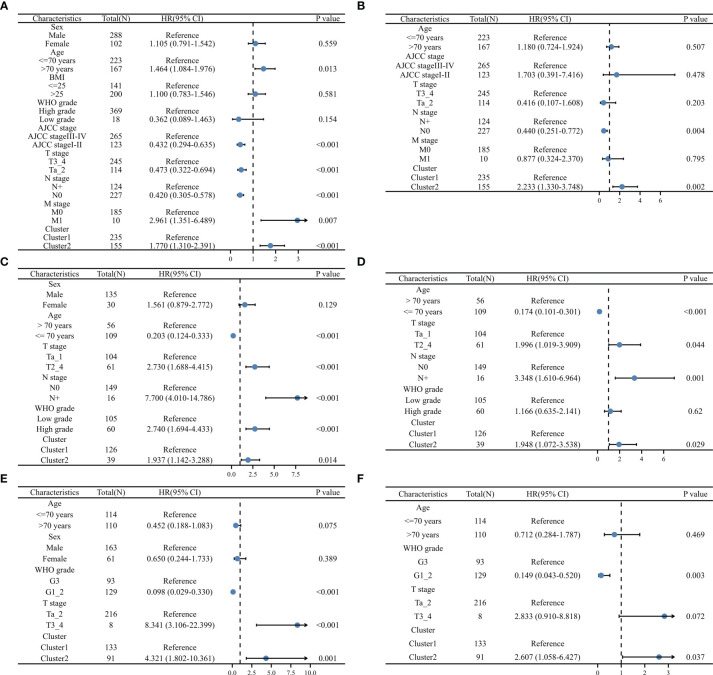
Validation of the independent prognostic value of the cluster: univariable **(A)** and multivariable **(B)** Cox regression model in TCGA dataset, univariable **(C)** and multivariable **(D)** Cox regression model in GSE13507 dataset, univariable **(E)** and multivariable **(F)** Cox regression model in GSE32984 dataset, N, lymph node metastasis; M, distant metastasis; WHO, World Health Organization.

The univariable and multivariable Cox regression was analyses were utilized to evaluate the independent prognostic value of ERC. In the TCGA dataset, the multivariable Cox regression model consisted of age, AJCC stage, T stage, lymph node metastasis stage, distant metastasis stage and ERC (**Figure 4A**), identifying that the cluster presented independent prognostic value (**Figure 4B**, P=0.002). Similarly, in the GSE13507 dataset, the multivariable Cox regression model consisted of age, T stage, WHO grade and ERC (**Figure 4C**), which validated that the cluster had independent prognostic value (**Figure 4D**, P=0.029). In the GSE32894 dataset, the multivariable Cox regression model consisted of age, WHO grade and ERC (**Figure 4E**), which also identified that the cluster could independently predict the prognosis of BC patients (**Figure 4F**, P=0.037).

### The results of function analysis

**Figure 5:**
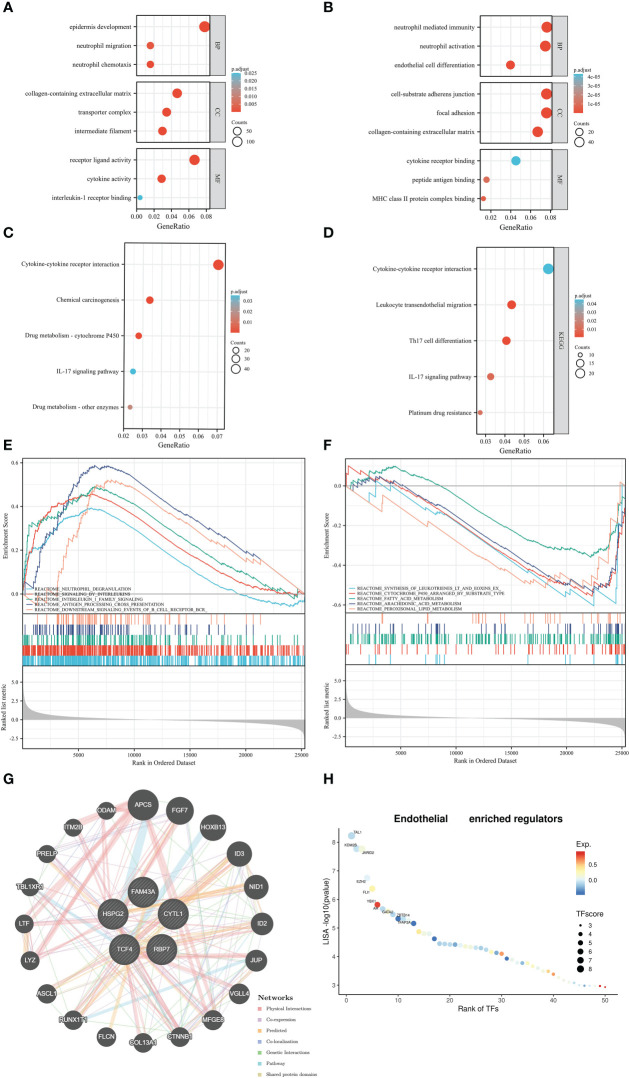
Function analysis: the Gene Ontology results based on the ERC **(A)** and endothelial cells **(B)**, Kyoto Encyclopedia of Genes and Genomes results based on the ERC **(C)** and endothelial cells **(D)**, Gene Set Enrichment Analysis results **(E, F)**, the protein–protein interaction network **(G)**, the interacting transcription factors of endothelial cells **(H)**.

As shown in **Figure 5A**, the ERC enriched immune-related and endothelial-related results, such as neutrophil chemotaxis, neutrophil migration, collagen-containing extracellular matrix, intermediate filament, receptor ligand activity, and cytokine activity in GO results. Based on endothelial cells, GO also enriched immune-related and endothelial-related results (**Figure 5B**). In KEGG analysis, ERC was predominantly implicated in immune-related and metabolism-related pathways (**Figure 5C**), including the IL-17 signaling pathway, cytokine−cytokine receptor interaction, and drug metabolism-cytochrome P450. Immune-related pathways also appeared in the enriched results of endothelial cell (**Figure 5D**). Interestingly, endothelial cell was involved in platinum drug resistance pathway. Similarly, the GSEA results also revealed enrichment of immune-related pathways (**Figure 5E**), such as neutrophil degranulation, antigen processing cross presentation, and downstream signaling events of BCR pathways. In **Figure 5F**, the ERC was additionally involved the process of metabolism, including fatty acid metabolism, arachidonic acid metabolism and peroxisomal lipid metabolism. According to the results of GeneMANIA, the interacting proteins of the ERC were APCS, FGF7, HOXB13, ID3 and others (**Figure 5G**). **Figure 5H** displayed the top interacting transcription factors of endothelial cell.

### The results of immune-related analysis

**Figure 6:**
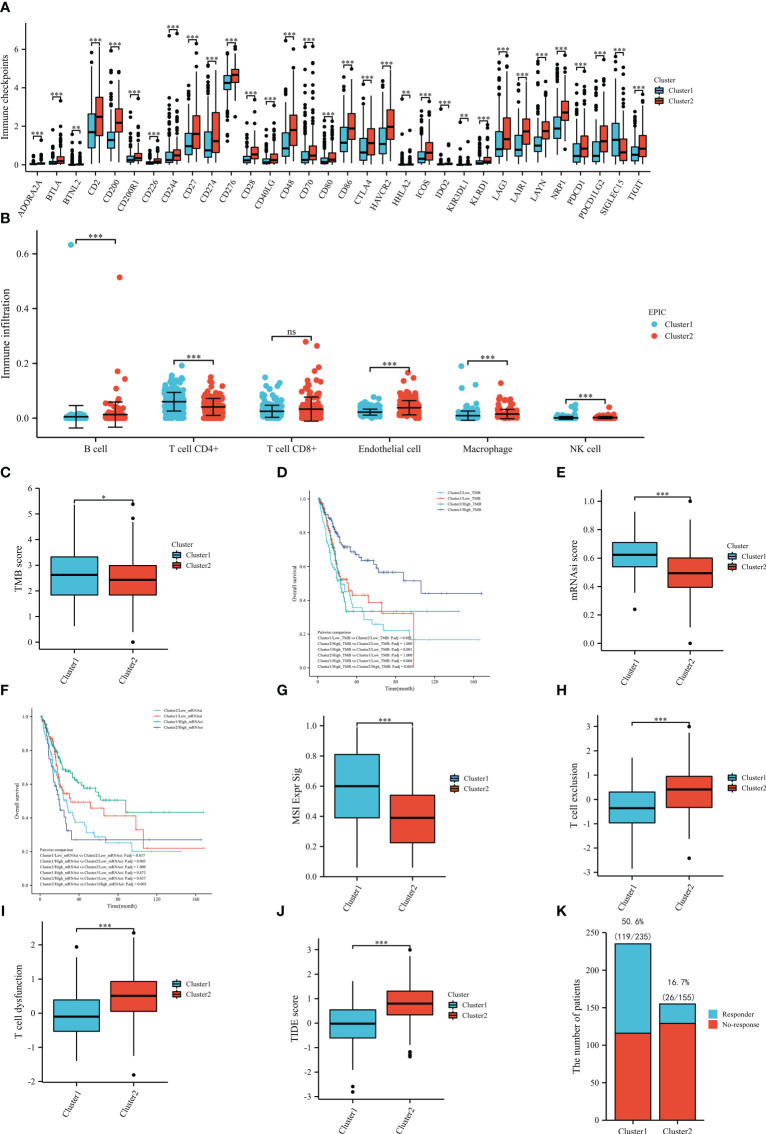
Immune-related analysis based on TCGA dataset: immune checkpoints **(A)**, immune infiltration **(B)**, the tumor mutational burden between the clusters **(C)**, the overall survival in groups **(D)**, the stemness index between the clusters **(E)**, the overall survival in groups **(F)**, the MSI Expr Sig score in the clusters **(G)**, the T-cell exclusion score between the clusters **(H)**, the T-cell dysfunction score between the clusters **(I)**, the TIDE score between the clusters **(J)**, the number of the patients with or without response to immunotherapy between the clusters **(K)**. TMB, tumor mutational burden; mRNAsi, stemness index. ns: P≥0.05; *, P< 0.05; **, P<0.01; ***, P<0.001.

Since the ERC enriched immune-related pathways, we decided to explore the role of the cluster in immune analysis. In immune checkpoints, cluster 2 was positively associated with the expression of many genes, such as CD274, CTLA4, PDCD1, PDCD1LG2, and LAG3. Only SIGLEC15 was highly expressed in the cluster 1 (**Figure 6A**). In the EPIC results, samples in the cluster 1 notably had a higher proportion of CD4+ T cell and NK cell infiltration, whereas samples in the cluster 2 infiltrated more B cells, endothelial cells and macrophages (**Figure 6B**).

After calculating the TMB score of each included sample in TCGA, we compared the scores between the clusters and identified that the mean score of cluster 1 was significantly higher than that of cluster 2, which might indicate that patients in the cluster 1 may be more likely to benefit from immunotherapy (**Figure 6C**). Then, we also compared the OS between the four subgroups and found that patients in the cluster 1 and high TMB score subgroups had the best prognosis (**Figure 6D**). To further pinpoint the above results, we compared the mRNAsi scores between the clusters. As shown in **Figure 6E**, the results of the stemness index illustrated that the cluster 1 had a statistically higher mRNAsi score than the cluster 2, which also supported that patients in the cluster 1 were more likely to benefit from immunotherapy. In the prognostic analysis, patients in the cluster 1 and high mRNAsi score subgroups had the best prognosis (**Figure 6F**). In terms of TIDE analysis, interestingly, cluster 1 was positively correlated with the MSI Expr Sig score (**Figure 6G**), while cluster 2 was positively related to T-cell exclusion (**Figure 6H**) and T-cell dysfunction (**Figure 6I**). Meanwhile, cluster 1 had a significantly lower TIDE score than cluster 2 (**Figure 6J**). In the prediction of immunotherapy, there were 119/235 (50.6%) responders in the cluster 1, while there were only 26/155 (16.7%) responders in cluster 2 (**Figure 6K**). All results of TIDE analysis supported that patients in the cluster 1 were more likely to benefit from immunotherapy. The correlation between endothelial cell and immunotherapy was evaluated by TIDE (**Supplementary Figure 1**).

## Discussion

In contrast to endothelial cells surrounded by pericytes in nonmalignant tissues, endothelial cells in the tumor vasculature have lower pericyte coverage and loose and leaky inter-endothelial cell junctions (8). Within tumor locations, This deficiency leads to hypoxia and insufficient nutrient supply (9). Furthermore, in the BC microenvironment, endothelial cells can induce poor prognosis by releasing von Willebrand Factor (VWF) (10). Given the important role of endothelial cells in the tumor microenvironment, we orchestrated molecular subtypes and identified key genes for BC from the perspective of endothelial cells, laying the possibility for developments in precision medicine. In this study, for the first time, we were able to classifiy and identify distinctly prognosis-related molecular subtypes at the genetic level by integrating single-cell and bulk RNA sequencing data. Meanwhile, we explored the correlation between the clusters and immunotherapy.

Cytokine-like protein 1 (CYTL1), located on human chromosome 4p15-p16, is a protein coding gene, which has proangiogenic effects *via* promoting sprouting and vessel formation (19, 20). Family with sequence similarity 43 member A (FAM43A) is a protein coding gene and can predict the prognosis of triple-negative breast cancer (21). Heparan Sulfate Proteoglycan 2 (HSPG2), encoding the perlecan protein, has an anti-angiogenesis effect and interacts with VEGFR2 on the surface of endothelial cells (22, 23). Retinol Binding Protein 7 (RBP7) can promote the migration and invasion of colon cancer cells (24). Transcription factor 4 (TCF4), a member of the helix–loop–helix (bHLH) transcription factor family, is upregulated in BC tissues and may serve as a predictor for BC patients (25). These results indicated that these five genes were involved in the function of endothelial cells in the tumor microenvironment or in the development of BC.

When stimulated by BC cell-derived small extracellular vesicles, endothelial cells can enhance O-GlcNAcylation to promote angiogenesis in BC tissues (26). Furthermore, endothelial cells can be activated by interleukin (IL)-1 to facilitate BC progression and dissemination (27). Using CD34 as a marker for endothelial cells, three studies confirm that endothelial cells can predict prognosis in BC patients (28–30). These results indicate that endothelial cells can significantly influence the development of BC. Consistent with the aforementioned literature, in our study, patients with high endothelial cell infiltration were statistically associated with worse OS than those with low endothelial cell infiltration according to the results of EPIC in the TCGA dataset. This result might be due to endothelial cell-induced tumor progression and metastasis. In the ERC, patients in the cluster 2 were positively correlated with endothelial cell infiltration and had considerably poorer OS, which was consistent with previous studies. Meanwhile, patients with BC appeared to benefit independently from the ERC. Hence, we propose that ERC may be able to forecast patients with BC’s prognosis.

According to the functional analysis’s findings, the ERC was enriched particularly rich in metabolic, endothelial-related and metabolism-related pathways. Meanwhile, the results of endothelial cell enrichment were congruent with the results of ERC, suggesting that the ERC was genuinely built on endothelial-related genes. The GO results, including collagen-containing extracellular matrix and intermediate filaments, revealed that ERC was associated with endothelial cells. GSEA of enriched pathways, including fatty acid metabolism, arachidonic acid metabolism, and peroxisomal lipid metabolism, identified the ERC was involved in the regulation of metabolism. In accordance with the present results, previous studies have demonstrated that BC cells’ metabolism is rewired as a result of hypoxia caused by endothelial cells in the tumor vasculature, which increases the invasive capabilities of the cells (13). The interaction protein FGF7 is expressed only by epithelial cells, indicating that the constructed ERC genes were closely related to endothelial cells. Another interaction protein, HOXB13, could participate in various immune-associated processes, which was consistent with the functional results of the ERC.

We explored the correlation between the ERC and immune analysis due to both functional analysis and interaction proteins which indicated that the cluster was involved in immune-related processes. In immune checkpoints, cluster 2 was positively associated with the expression of numerous genes, such as CD274, CTLA4 and PDCD13, while only SIGLEC15 was highly expressed in the cluster 1. Due to the current controversy about the expression of immune checkpoints in BC and their predictive value for immunotherapy response, we were unable to make any recommendations on which cluster was more likely to benefit from immunotherapy (31). Only the fact that the clusters were at least different is known. Therefore, we assessed and compared the infiltrating cells between the clusters. Samples in the cluster 1 had a statistically significant increase in CD4+ T cells and NK-cell infiltration. CD4 T cells can enhance antitumor immunity by inhibiting angiogenesis (32). NK cells are suppressed in a hypoxic environment (33). This might have caused the high NK-cell infiltration in the cluster 1. Furthermore, high NK-cell infiltration was associated with a better immunotherapy response than low NK-cell infiltration (33). There was no discernible change in CD8 + T cell infiltration between the cluster 1 and cluster 2. This phenomenon might be explained by the results of TIDE analysis. The T cells in the cluster 2 were substantially related with a higher proportion of exclusion and dysfunction than T cells in the cluster 1. As we know, T cells with sufficient function are the cornerstone of immunotherapy response. Therefore, these results suggested that although the absolute number of CD8 + T cells in the clusters was equal, the cluster 1 might has more sufficient functional CD8 + T cell infiltration. Furthermore, in the cluster 2, more B cells infiltrated this cluster. Exhausted or dysfunctional CD8+ and CD4+ T cells are frequently programmed to solicit B-cell help in the face of tumor persistence (34). This fact might explain why the cluster 2 had more B-cell infiltration and identified the presumption that the cluster 1 might has more sufficient functional CD8 + T cell infiltration. Therefore, we hypothesized that the cluster 1 would be more likely to benefit from immunotherapy. Then, to further pinpoint the possibilities, we calculated and compared the TMB score between the two clusters. The mean TMB score of cluster 1 was substantially higher than that of cluster 2. A high TMB score was positively associated with the immunotherapy response rate, which also supported our hypotheses (35). Inadequate nutrient supply and hypoxia caused by inadequate nutrient supply and hypoxia promote enrichment of cancer stem cells (12, 36). Thus, we assessed the cancer stem cells in the two clusters by the mRNAsi score. A high mRNAsi score was positively associated with the immunotherapy response rate (17). Cluster 1 had a statistically higher mRNAsi score than cluster 2, which also supported that patients in the cluster 1 were more likely to benefit from immunotherapy. Given the results of TMB and mRNAsi, patients with high microsatellite instability (MSI) scores could benefit more from immunotherapy (35). The comparison results of the MSI Expr Sig score between the clusters supported that patients in the cluster 1 were more likely to benefit from immunotherapy. Notably, cluster 2 was positively related to T-cell exclusion and dysfunction. This result might explain why patients in the cluster 1 were more likely to respond favorably to immunotherapy, despite the fact that CD8+ T-cell infiltration between the two clusters. Furthermore, cluster 1 outperformed cluster 2 in terms of TIDE score, which indicated that cluster 1 might have a higher response rate than cluster 2. As a result, we displayed the response rate of these two clusters in **Figure 6K**. The results showed that 50.6% (119/235) of patients in the cluster 1 responded to immunotherapy, while the response rate in the cluster 2 decreased to 16.7% (26/155). According to these results, we might infer that patients in the cluster 1 were more likely to benefit from immunotherapy. Naturally, more *in vivo* and *in vitro* research on this discovery is required.

In the era of precision medicine, how to treat patients effectively, quickly, and affordably is a key concern. In this investigation, we identified molecular subtypes that could well predict the pathogenesis of individual patients with BC and predict the response to immunotherapy. In this approach, timely warning can be given from a clinical perspective before the dilemma of insufficient treatment and poor prognosis in patients with BC.

## Conclusion

In this study, we classified and identified distinctly prognosis-related molecular subtypes and key genes from the perspective of endothelial cells at the genetic level by integrating single-cell and bulk RNA sequencing data, primarily to provide a roadmap for precision medicine.

## Data availability statement

All data from this study were downloaded from an online database. Therefore, everyone can get the data online. Further inquiries can be directed to the corresponding author.

## Author contributions

All authors contributed to the study conception and design. Interpretation of data was performed by D-XL, D-CF, XS, R-CW, KC, PH. The first draft of the manuscript was written by D-XL, XS, and all authors commented on previous versions of the manuscript. The final version of the manuscript was written by XS, D-CF, and PH. All authors contributed to the article and approved the submitted version. 

## Supplementary material

Supplementary Figure 1 The correlation between TIDE score and endothelial cells **(A)**, the MSI Expr Sig score in the high and low endothelial cells **(B)**, the T-cell exclusion score between the high and low endothelial cells **(C)**, the T-cell dysfunction score between the high and low endothelial cells **(D)**, the number of the patients with or without response to immunotherapy betwee the high and low endothelial cells **(E)**.
