# Supplementary figures and images for: Identification of endothelial-related molecular subtypes for bladder cancer patients
Source: Front Oncol. 2023 Mar 21;13:1101055. doi: 10.3389/fonc.2023.1101055 (PMC10070733; doi:10.3389/fonc.2023.1101055)

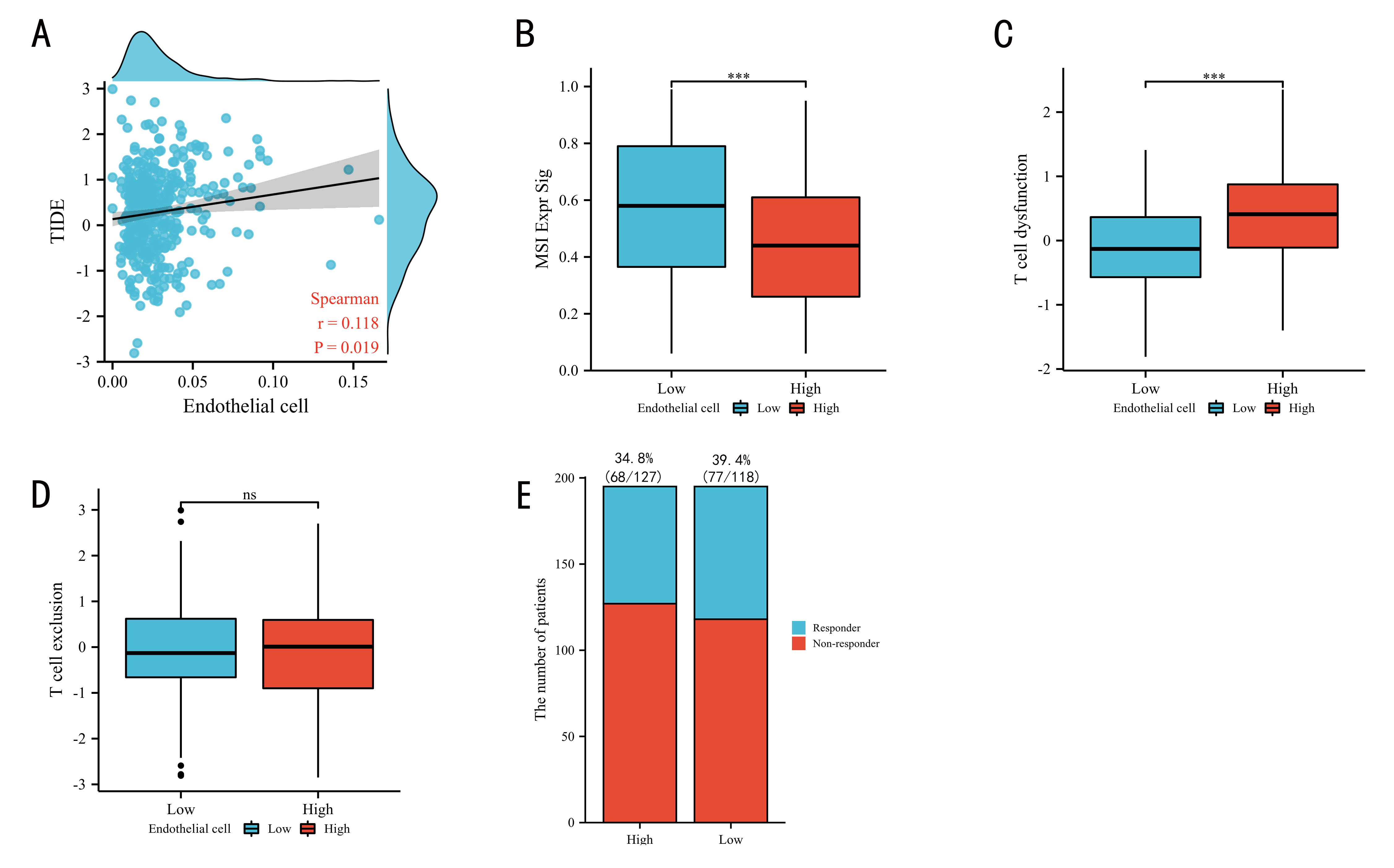

Supplement: Supplementary Figure 1 — The correlation between TIDE score and endothelial cells (A), the MSI Expr Sig score in the high and low endothelial cells (B), the T-cell exclusion score between the high and low endothelial cells (C), the T-cell dysfunction score between the high and low endothelial cells (D), the number of the patients with or without response to immunotherapy betwee the high and low endothelial cells (E). [file Image_1.jpeg]
