# Supplementary material for: Identification of endothelial-related molecular subtypes for bladder cancer patients
Source: Front Oncol. 2023 Mar 21;13:1101055. doi: 10.3389/fonc.2023.1101055 (PMC10070733; doi:10.3389/fonc.2023.1101055)
Supplement: Supplementary file 3 [file Table_2.docx]

Supplementary table 2. The Clinicopathologic characteristics of the GSE13507 included patients.

| Characteristic | Cluster1 | Cluster2 | p |
| --- | --- | --- | --- |
| n | 126 | 39 |  |
| Age, mean ± SD | 65.4 ± 12.61 | 64.49 ± 9.72 | 0.680 |
| Sex, n (%) |  |  | 0.779 |
| Female | 24 (14.5%) | 6 (3.6%) |  |
| Male | 102 (61.8%) | 33 (20%) |  |
| T stage, n (%) |  |  | < 0.001 |
| T2_4 | 34 (20.6%) | 27 (16.4%) |  |
| Ta_1 | 92 (55.8%) | 12 (7.3%) |  |
| Lymph node metastasis, n (%) |  |  | 0.062 |
| N+ | 9 (5.5%) | 7 (4.2%) |  |
| N0 | 117 (70.9%) | 32 (19.4%) |  |
| WHO grade, n (%) |  |  | 0.043 |
| High grade | 40 (24.2%) | 20 (12.1%) |  |
| Low grade | 86 (52.1%) | 19 (11.5%) |  |
| Overall survival, n (%) |  |  | 0.236 |
| Alive | 77 (46.7%) | 19 (11.5%) |  |
| Dead | 49 (29.7%) | 20 (12.1%) |  |

SD: Standard deviation; WHO: World Health Organization; n: Number.
