# Supplementary material for: Identification of endothelial-related molecular subtypes for bladder cancer patients
Source: Front Oncol. 2023 Mar 21;13:1101055. doi: 10.3389/fonc.2023.1101055 (PMC10070733; doi:10.3389/fonc.2023.1101055)
Supplement: Supplementary file 4 [file Table_3.docx]

Supplementary table 3. The Clinicopathologic characteristics of the GSE32894 included patients.

| Characteristic | Cluster1 | Cluster2 | p |
| --- | --- | --- | --- |
| n | 133 | 91 |  |
| Age, mean ± SD | 68.77 ± 12.66 | 70.41 ± 8.87 | 0.256 |
| Sex, n (%) |  |  | 1.000 |
| Female | 36 (16.1%) | 25 (11.2%) |  |
| Male | 97 (43.3%) | 66 (29.5%) |  |
| WHO grade, n (%) |  |  | < 0.001 |
| G1_2 | 91 (41%) | 38 (17.1%) |  |
| G3 | 42 (18.9%) | 51 (23%) |  |
| T stage, n (%) |  |  | 0.275 |
| T3_4 | 3 (1.3%) | 5 (2.2%) |  |
| Ta_2 | 130 (58%) | 86 (38.4%) |  |
| Overall survival, n (%) |  |  | 0.002 |
| Alive | 126 (56.2%) | 73 (32.6%) |  |
| Dead | 7 (3.1%) | 18 (8%) |  |

SD: Standard deviation; WHO: World Health Organization; n: Number.
